# Supplementary material for: Engineering nanowires in bacteria to elucidate electron transport structural–functional relationships
Source: Sci Rep. 2023 May 31;13:8843. doi: 10.1038/s41598-023-35553-2 (PMC10232502; doi:10.1038/s41598-023-35553-2)
Supplement: Supplementary file 1 — Supplementary Information. [file 41598_2023_35553_MOESM1_ESM.docx]

**Engineering Nanowires in Bacteria to Elucidate Electron Transport Structural-Functional Relationships**

**Supporting Information**

Ben Myers^1,3^, Francesco Catrambone^2^, Stephanie Allen^3^, Phil J Hill^4^, Katalin Kovacs^1,3*^, Frankie J Rawson^1*^

* Corresponding authors Frankie.Rawson@nottingham.ac.uk, Katalin.Kovacs@nottingham.ac.uk

^1^ Bioelectronics Laboratory, Regenerative Medicine and Cellular Therapies, School of Pharmacy, Biodiscovery Institute, The University of Nottingham, Nottingham, NG7 2RD, UK

^2^ BBSRC/EPSRC Synthetic Biology Research Centre, School of Life Sciences, Biodiscovery Institute, University of Nottingham, NG7 2RD, United Kingdom

^3^ Molecular Therapeutics and Formulation Division, School of Pharmacy, Boots Science Building, University Park, The University of Nottingham, Nottingham, NG7 2RD, UK

^4^ Division of Microbiology, Brewing and Biotechnology, School of Biosciences, Sutton Bonington Campus, University of Nottingham, LE12 5RD, United Kingdom

**Supplementary Methods:**

| Plasmid | Function | Source |
| --- | --- | --- |
| pMTL70641:SacB3 | Suicide plasmid host vector | Muhammad Ehsaan ^1^ |
| pMTL70641:SacB3:PilAKO | Suicide plasmid (PilA deletion) | This study |
| pMTL71301: araBAD | MPilA expression host vector | Muhammad Ehsaan ^1^ |
| pMTL71301:araBAD:mPilA:Empty | Modified PilA negative control (no MpilA cargo) | This study |
| pMTL71301:araBAD:mPilA:T80 | Modified PilA expression (strain T80) | This study |
| pMTL71301:araBAD:mPilA:T61 | Modified PilA expression (strain T61) | This study |
| pMTL71301:araBAD:mPilA:T61Y | Modified PilA expression (strain T61Y) | This study |
| pMTL71301:araBAD:mPilA:T61W | Modified PilA expression (strain T61W) | This study |

**Table S1:** Table of plasmids used in this study.

**Table S2:** Table of primers used in this study.

| Primer | Sequence (5”-3”) | Function |
| --- | --- | --- |
| HIFI:PilAKO:UpHA:FWD | TTCGAGCTCGGTACCCGGGGATCCTCTTCCACCACATTCTGGATTG | Amplification/ HIFI Assembly |
| HIFI:PilAKO:UpHA:REV | GGCATTCCGTACGATTTGACCCCTCGAAGAG | Amplification/ HIFI Assembly |
| PilAKO:DownHA:FWD | GAGGGGTCAAATCGTACGGAATGCCTGAGAAAAAGCGCCTC | Amplification/ HIFI Assembly |
| PilAKO:DownHA:REV | TGCCAAGCTTGCATGTCTGCAGGCCTGCGCCGCGTGCGGACAG | Amplification/ HIFI Assembly |
| Diag:PilAKO:Ext:FWD | TTCCAGCGAGCGCTGGAATGGCGAA | PCR Diagnostics |
| Diag:PilAKO:Ext:REV | GACTTGCTGCAACTGCTGGAATTGCGC | PCR Diagnostics |
| Seq:PilAKO:Up:FWD | CTTCCACCACATTCTGGATTG | Sequencing |
| Seq:PilAKO:Int:FWD | ATGCAACGGGTACAACAACTG | Sequencing |
| Seq:PilAKO:Down:REV | GATAGCGAAGAGCGCAAGCA | Sequencing |
| HiFi:71301:MPilA:ALL:FWD | CGACGTCACGCGTCCATGGAACCGCGGCCGCTGTCAAA | MPilA Insert HiFi Assembly |
| HiFi:71301:MPilA:ALL:REV | CGATGACGACAAGTAATAAGCGCTAGCATTGGCACTGGCCGTCGTTTTA | MPilA Insert HiFi Assembly |
| Diag:71301:MPilA:All:FWD | CGCTTCAGCCATACTTTTCATAC | PCR Diagnostics/ Sequencing |
| Diag:71301:MPilA:All:REV | GCTCTTGGATGGAGGAAATGAC | PCR Diagnostics/ Sequencing |

|  | **Oxidative Peak Current (µA)**  **I_pa_** | **Oxidative Peak**  **Potential (V)**  **E_pa_** | **Reductive Peak Current (µA)**  **I_pc_** | **Reductive Peak**  **Potential (V)**  **E_pc_** |
| --- | --- | --- | --- | --- |
| **WT** | 0.028 (+/- 0.029) | 0.017 (+/- 0.03) | -0.025 (+/- 0.035) | 0.075 (+/-0.23) |
| **ΔpilA** | 0.023 (+/- 0.01) | 0.2 (+/- 0.0) | -0.015(+/- 0.03) | 0.05 (+/- 0.05) |
| **T80** | 0.062 (+/- 0.041) | 0.17 (+/- 0.06) | -0.03 (+/- 0.02) | -0.05 (+/- 0.08) |
| **T61** | 0.04 (+/- 0.03) | 0.23 (+/- 0.058) | -0.039 (+/- 0.02) | 0.03 (+/- 0.058) |
| **T61Y** | 1: 0.23 (+/- 0.157)  2: 0.15 (+/- 0.05) | 1: -0.22 (+/- 0.029)  2: 0.2 (+/- 0.1) | -0.05 (+/- 0.038) | 0.2 (+/- 0.09) |
| **T61W** | 0.26 (+/- 0.11) | 0.1 (+/- 0.1) | -0.11 (+/- 0.1) | 0.02 (+/- 0.11) |

**Table S3:** Mean peak size and potential of biofilm- modified electrodes, detected via cyclic voltammetry exemplified in figure 5 of the main manuscript.

**Table S4:** Cytochrome BLASTp sequence alignment within all non-redundant GenBank CDS translations + PDB + SwissProt + PIR + PRF excluding environmental samples from WGS projects databases. Queried microorganisms: *G. sulfurreducens* KN400 (taxid:663917), *Geobacter sulfurreducens* (strain ATCC 51573), *Shewanella oneidensis* MTR-1, *Rhodopseudomonas palustris* TIE-1, *C. necator* H16 (taxid:381666).

| **Description** | **Scientific name / Taxid** | **Max score** | **Query cover** | **E value** | **Identity (%)** | **Acc. Length** | **Gene ID:** |
| --- | --- | --- | --- | --- | --- | --- | --- |
| OmcZ | *Geobacter sulfurreducens* (strain ATCC 51573) | No significant similarity found | n/a | n/a | n/a | 473 | Q74BG5 |
| OmcS | *Geobacter sulfurreducens* (strain ATCC 51573) | No significant similarity found | n/a | n/a | n/a | 432 | Q74A86 |
| OmcE | *Geobacter sulfurreducens* (strain ATCC 51573) | No significant similarity found | n/a | n/a | n/a | 232 | Q74FJ0 |
| OmcT | *Geobacter sulfurreducens* (strain ATCC 51573) | No significant similarity found | n/a | n/a | n/a | 430 | Q74A87 |
| OmcF | *Geobacter sulfurreducens* (strain ATCC 51573) | No significant similarity found | n/a | n/a | n/a | 104 | Q74AE4 |
| mtrC | *Shewanella Oneidensis* MTR-1 | No significant similarity found | n/a | n/a | n/a | 671 | Q8EG34 |
| omcA | *Shewanella Oneidensis* MTR-1 | No significant similarity found | n/a | n/a | n/a | 735 | Q8EG33 |
| PioA | *Rhodopseudomonas palustris* TIE-1 | No significant similarity found | n/a | n/a | n/a | 540 | A1EBT2-1 |
| MtoA | *Sideroxydans lithotrophicus ES-1* | 47.8 | 45% | 3e-08 | 42.11 | 125 | D5CN26 |
| c-type cytochrome | *Cupriavidus necator* H16 (381666) | 47.8 | 45% | 3e-08 | 42.11 | 220 | WP_010813388.1 |
| PgcA | *Geobacter sulfurreducens* (strain ATCC 51573) | 32.7 | 6% | 0.033 | 38.71 | 511 | Q74CB3 |
| Cytochrome c family protein | *Cupriavidus necator* H16 (381666) | 32.7 | 6% | 0.033 | 38.71 | 125 | WP_011617786.1 |


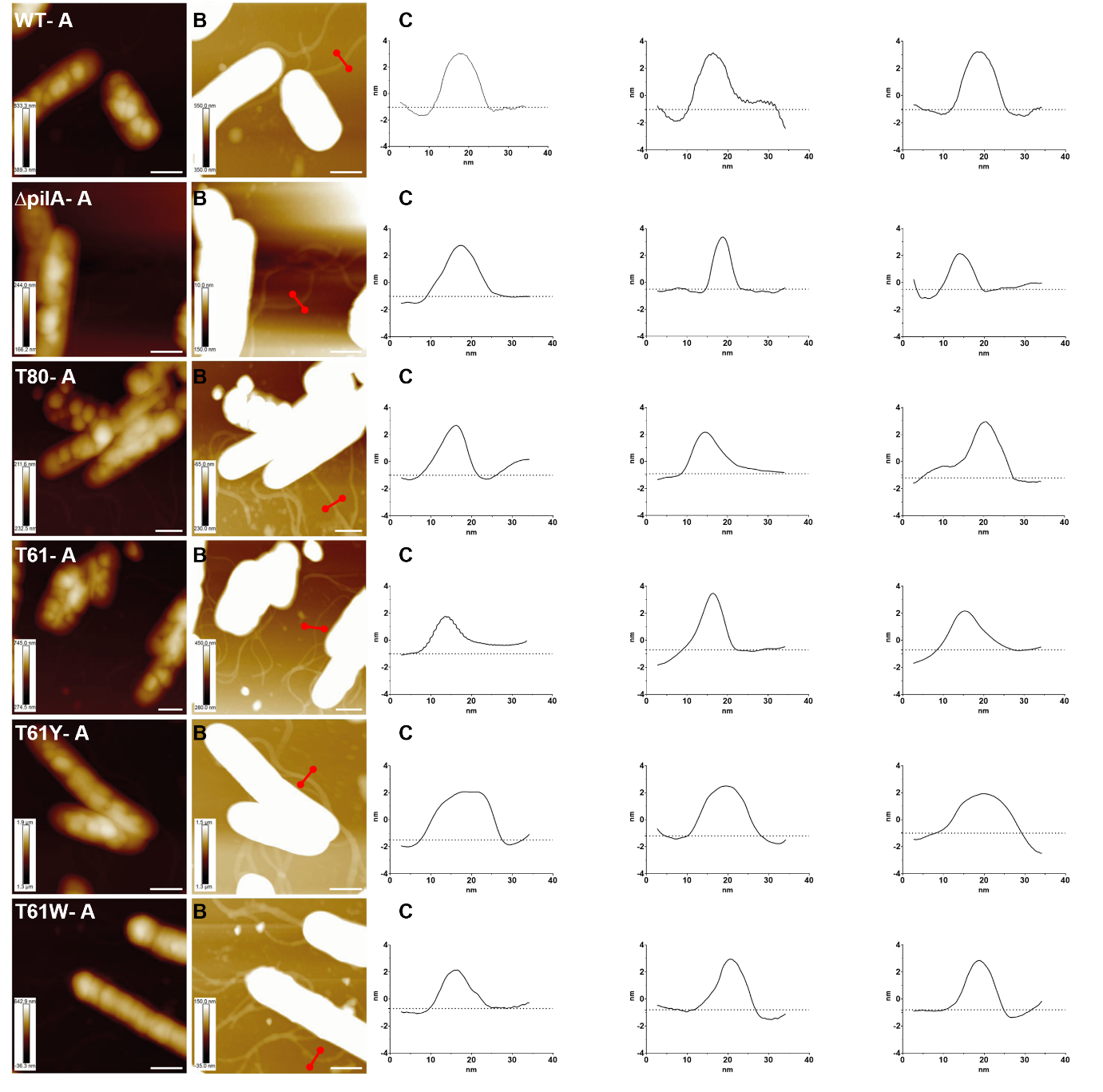


**Figure S1: Analysis of AFM topographical data to determine pili morphology.** A: Raw topographical (height) data map of *CN* strains WT- T61W, with z- scale automatically determined via Nanoscope analysis software. Due to large height differential between bacterial cells and filaments, nanoscale features are difficult to observe in the raw z- scale and so manual narrowing was required. B: Topographical data map with z- scale manually decreased to bring features within range to allow visualisation. Z- scale shown as insert in bottom- left, and white bar in bottom right representing a lateral scale of 1 µm in both A and B. Red cross sectional bar indicating representative locations for height profile gradient analysis in B. C: Line profile analysis of pili- cross sectional height, generated via Gwyddion. Each graph displays the mean of three measurements, taken across an individual filament. Three filaments were analysed for each strain, making a total n of 9 measurements. The baseline used to calculate filament height is displayed as the dotted line within each graph. All variants had a mean filament morphology of 4.5-5.7nm (WT: 5.4nm SD ± 0.4, Δ*pilA*: 5.3nm SD ± 0.41, T80: 4.8nm SD ± 0.55, T61: 4.5nm SD ± 0.8, T61Y: 5.7nm SD ± 0.8, WT: 5.6nm SD ± 0.8. Differences in recombinant pili morphology were not significant (p>0.05), based on one- way ANOVA analysis of control pilus (WT) compared to experimental (Δ*pilA, T80, T61, T61Y, T61W)*. Data analysis performed via Graphpad prism.


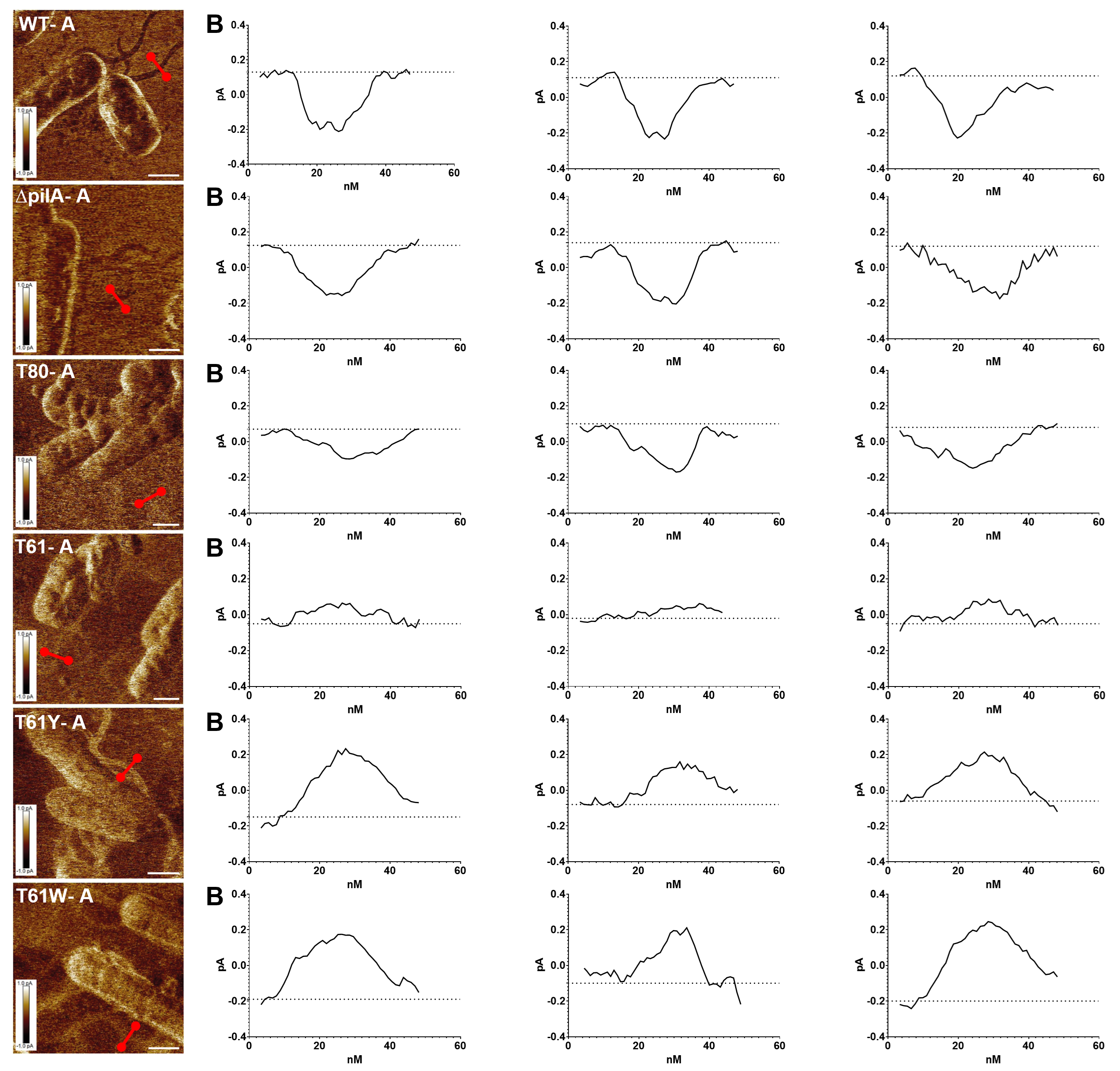


**Figure S2. AFM Contact Current data analysis to determine current profile of pili.** A: Contact current data map of *CN* strains WT- T61W. Z- scale shown as insert in bottom- left, aligned across all data maps to a scale of +1pA to -1pA and white bar in bottom right representing a lateral scale of 1 µm. Red cross sectional bar indicating representative locations for contact current profile gradient analysis. B: Contact current line profile gradients for *CN* strains WT- T61W respectively. Each graph displays the mean of three measurements taken across an individual filament. Three filaments were analysed for each strain, making a total n of 9 measurements. Line profile analysis of pili- cross sectional contact current generated via Gwyddion^2^. The baseline used to calculate filament conductivity is displayed as the dotted line within each graph. WT:-0.33pA SD ± 0.01; ΔpilA:-0.31pA SD ± 0.03; ΔpilA:mPilAT80: 0.21pA SD ± 0.05; ΔpilA:mPilAT61: 0.09pA SD ± 0.03; ΔpilA:mPilAT61Y: 0.29pA SD ± 0.08; ΔpilA:mPilAT61W: 0.36pA SD ± 0.06. Data analysis performed via GraphPad Prism.

**
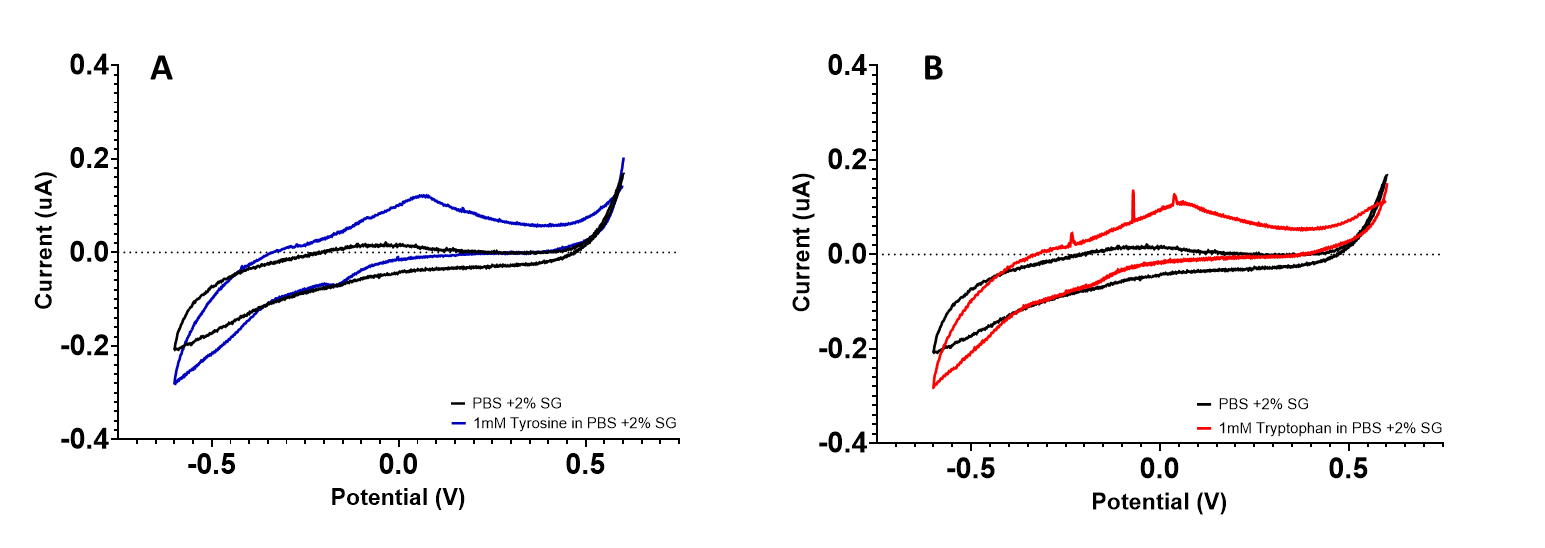
Figure S3: Cyclic voltammetry analysis of abiotic amino acid solutions**. A: 1mM tyrosine in PBS + 2% sodium gluconate (blue) B: 1mM tryptophan in PBS + 2% sodium gluconate (red). Both are compared to bare PBS + 2% sodium gluconate only (A, B: black trace). Scans were performed from a starting potential of 0.6V to -0.6V, at a scan rate of 1mVs.

**References:**

1. Ehsaan, M., Baker, J., Kovács, K., Malys, N. & Minton, N. P. The pMTL70000 modular, plasmid vector series for strain engineering in *Cupriavidus necator* H16. *J. Microbiol. Methods* **189**, 9–11 (2021).

2. Nečas, D. & Klapetek, P. Gwyddion: An open-source software for SPM data analysis. *Cent. Eur. J. Phys.* **10**, 181–188 (2012).
